# Amplitude modulated transcranial alternating current stimulation (AM-TACS) efficacy evaluation via phosphene induction

**DOI:** 10.1038/s41598-021-01482-1

**Published:** 2021-11-15

**Authors:** Carsten Thiele, Tino Zaehle, Aiden Haghikia, Philipp Ruhnau

**Affiliations:** 1 Department of Neurology, Otto-Von-Guericke-Universität, Universitätsklinikum Magdeburg, Leipziger Str. 44, 39120 Magdeburg, Germany; 2 Center for Behavioral Brain Sciences (CBBS), Otto-Von-Guericke-Universität Magdeburg, 39120 Magdeburg, Germany; 3 German Center for Neurodegenerative Diseases, Magdeburg, Germany

**Keywords:** Neuroscience, Psychology

## Abstract

Amplitude modulated transcranial alternating current stimulation (AM-tACS) is a novel method of electrostimulation which enables the recording of electrophysiological signals during stimulation, thanks to an easier removable stimulation artefact compared to classical electrostimulation methods. To gauge the neuromodulatory potential of AM-tACS, we tested its capacity to induce phosphenes as an indicator of stimulation efficacy. AM-tACS was applied via a two-electrode setup, attached on FpZ and below the right eye. AM-tACS waveforms comprised of different carrier (50 Hz, 200 Hz, 1000 Hz) and modulation frequencies (8 Hz, 16 Hz, 28 Hz) were administered with at maximum 2 mA peak-to-peak stimulation strength. TACS conditions in the same frequencies were used as a benchmark for phosphene induction. AM-tACS conditions using a 50 Hz carrier frequency were able to induce phosphenes, but with no difference in phosphene thresholds between modulation frequencies. AM-tACS using a 200 Hz or 1000 Hz carrier frequency did not induce phosphenes. TACS conditions induced phosphenes in line with previous studies. Stimulation effects of AM-tACS conditions were independent of amplitude modulation and instead relied solely on the carrier frequency. A possible explanation may be that AM-tACS needs higher stimulation intensities for its amplitude modulation to have a neuromodulatory effect.

## Introduction

Neuronal oscillations across a range of frequencies are the basis for communication in the brain and underly many cognitive functions^1^. Transcranial alternating current stimulation (tACS) allows us to modulate this oscillatory activity in the brain^2–6^. On single-neuron level, tACS causes shifts in frequency and phase of neuronal spike timing^7^. On a population level, it is thought that tACS leads to “entrainment”, i.e., phase-locking of neuronal activity to the applied frequency^8^. When applied at a task-relevant frequency, tACS therefore can lead to observable changes in behaviour (and presumably change in underlying function), due to modulations of task-relevant brain oscillations^5^. Because of that, tACS has been used to research many different brain functions, including memory^9^, motor performance^10^, working memory^11^, creative thinking^12^ or motion perception^13^, just to name a few.

Most tACS studies are focussing on the behavioural effects of stimulation^14–16^, while they lack electrophysiological recordings to confirm the modulation of neuronal activity. This is due to a significant artefact to any electrophysiological recording caused by the electrostimulation which poses a challenge to analyse online-effects (i.e., effects during stimulation) of tACS^17^. Other studies bypass this problem by relying on offline-effects (i.e., effects after stimulation) of tACS. Previous studies were able to demonstrate modulations of endogenous oscillations after tACS which outlast the stimulation for up to 70 min^18^ (for a review see^19^). However, offline-effects are likely not based on neuronal entrainment but rather on changes in neuronal plasticity, therefore it has been concluded that online- and offline-effects are qualitatively different^2^.

To be able to study online-effects, it is necessary to analyse (artefact-free) brain activity recorded during stimulation. This has been proven very challenging, since signal subtraction approaches may be able to reduce the amplitude of linear stimulation artefacts by a significant amount^20^, but they fail to eliminate nonlinear stimulation artefacts introduced by, for instance, broadband noise or interactions of physiological processes (e.g., respiratory-, cardiac- or oculomotoric activity) with the stimulation^17^. There is typically a substantial amount of residual artefact which might be mistaken as brain activity^21^. A further complicating fact is that the stimulation frequency and neuronal frequency of interest are identical, making the use of spectral filters unfeasible to reduce artefact strength, since the brain activity of interest would be eliminated from the data as well. Therefore, more sophisticated methods are needed to recover the real brain signal from the overlapping stimulation artefact. One of the possible solutions that was found for this problem, was to apply spatial filters like beamforming^22,23^. But there is still some debate as to how effective these filters are at removing the stimulation artefacts, as well as non-linear interactions^17,24,25^.

A different approach to circumvent the artefact problem was proposed by Witkowski et al.^26^ who used amplitude modulated tACS (AM-tACS). This method uses a stimulation waveform that consists of two components: a high-frequency (> 150 Hz) sinusoidal carrier and a low-frequency (e.g., 10 Hz) amplitude modulation. When combined, the modulation signal leads to a sinusoidally rising and falling amplitude of the carrier signal, often referred to as the envelope, generating an amplitude modulated waveform. It is important to note that it is not the high-frequency carrier signal which stimulates neuronal activity, but rather the low-frequency amplitude modulation. Other stimulation methods which rely on the combination of a carrier signal and a stimulating component already exist, for instance, in the form of cross-frequency tACS^27^ where typically a continuous low-frequency carrier is combined with a high-frequency signal aligned to a certain phase of the carrier (e.g., a 6 Hz carrier combined with short gamma-frequency bursts^28^). But a carrier frequency this low already has a stimulation effect by itself, which AM-tACS avoids by employing a carrier frequency that is too high to have a neurostimulatory effect. This is because of low-pass properties of neuronal membranes which attenuate high-frequency stimulation^29,30^. The amplitude modulation frequency, on the other hand, is chosen to be low enough to be able to entrain neuronal activity^31,32^. Recent work theoretically confirms the stimulation effect of the amplitude modulation and therefore the feasibility of AM-tACS, for instance, using modelling^33^ or with hippocampal slices in-vitro^34^. Previous studies also found stimulation effects of AM-tACS in humans, as it e.g. disrupted performance in a working memory task^35^ or affected visual perception^36^.

AM-tACS aims to allow for the analysis of online stimulation effects, by theoretically avoiding the contamination of the recorded brain oscillations at the frequency of interest with a stimulation artefact^26^. When using AM-TACS, the recorded signal should only be contaminated by the carrier frequency, which is way beyond the frequency of interest. The frequency of the amplitude modulation on the other hand exhibits no spectral power^37^, thus not introducing an artefact into the signal. As a result, AM-tACS—with an appropriate low pass filter to eliminate the carrier—should allow for stimulation while concurrently recording artefact-free brain signals at the frequency of interest, therefore making online effects observable. In practice however, recent studies were able to demonstrate that due to nonlinear transfer characteristics of stimulation and recording hardware, some artefacts are still introduced in the electrophysiological recording that have to be accounted for^37^, but can be removed using noise reduction techniques^36^.

Of note, a promising new type of transcranial electric stimulation using temporal interference^38^ relies on a similar amplitude modulated signal. This method does not use an amplitude modulated signal that is emitted from the electrodes (which is the case with AM-tACS), but instead relies on two interfering alternating current fields. Due to the fact that the fields alternate with differing frequencies, they result in an amplitude modulated current at the point of interference, i.e., in the targeted brain area where the fields overlap. This way, this method may allow for non-invasive stimulation of deeper brain structures by generating a remote-target AM-tACS-like signal. Thus, evaluating AM-tACS can also help to advance our knowledge about the efficacy and mechanism of action of temporal interference stimulation.

In our study, we probed the efficacy of AM-tACS using visual phosphenes. Phosphenes are perceptions of flashing or shimmering light in the absence of accompanying visual input which can be easily induced by applying an electric current to the retina^39^. Since the eye, or rather the retina, can be considered part of the central nervous system (CNS) and a model of electric CNS circuitry, retinal phosphenes have been used as a robust indicator to gauge efficacy of CNS stimulation^40^. Though electrically induced phosphenes have initially thought to be created by visual cortex stimulation^41^, it is now widely accepted that the electric current is travelling along the skin of the head to the eyes, causing retinal activation and thus making the retina the source of phosphenes^39,42–47^. In previous AM-TACS studies^35,36^, behavioral effects have been found, but subjects reported having seen no phosphenes. This is likely due to electrode placements targeting the brain, leading to low current density at the retina. Therefore, in our study we optimized the electrode setup to increase electric currents reaching the retina in order to maximize the phosphene induction potential.

**Figure 1:**
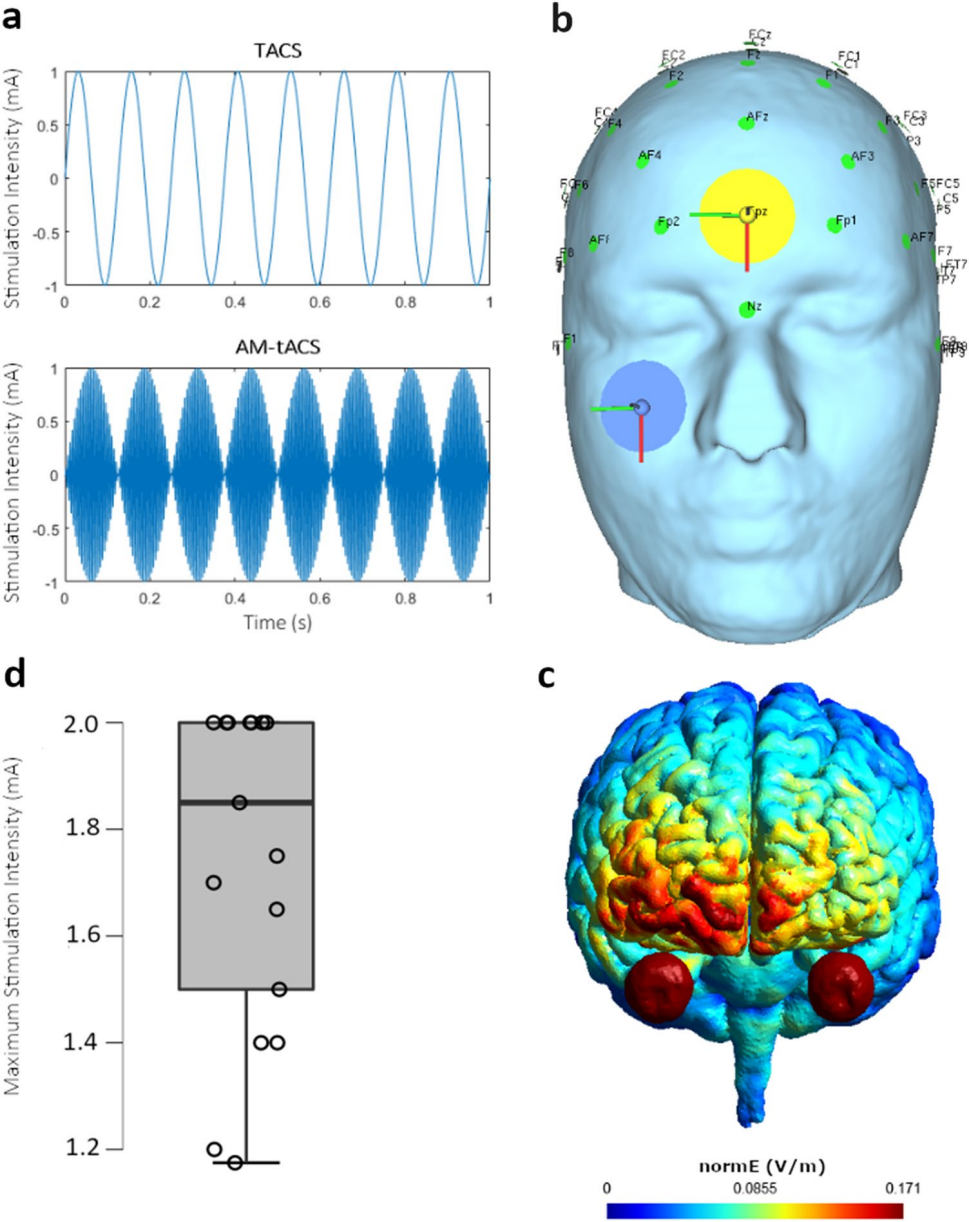
**(a)** Used stimulation methods exemplified. Top: TACS with 8 Hz. Bottom: AM-tACS with 8 Hz amplitude modulation frequency and 200 Hz carrier frequency. **(b)** Electrode setup used in this experiment with two 34 mm circular electrodes, placed at FpZ and on the cheek below the right eye. **(c)** Simulation of current distribution during 2 mA stimulation. Generated with SimNIBS ^50^. **(d)** Distribution of subjects’ individual maximum stimulation intensities as determined by the pain threshold procedure.

In our study, we examine the efficacy of AM-tACS (see Fig. 1a (Bottom)) stimulation on the CNS. This will be further evaluated by also applying classical tACS (see Fig. 1a (Top)) as a benchmark of stimulation efficacy. Phosphene thresholds (lowest needed stimulation intensity to induce phosphenes) will be used to compare stimulation efficacy of stimulation methods and -frequencies. In case of AM-tACS, we hypothesize that phosphene thresholds will overall vary between carrier frequencies, as the required stimulation intensity for an effective stimulation rises with higher carrier frequencies^33^. We further hypothesize that phosphene thresholds will vary within a carrier frequency, as a function of amplitude modulation frequency. This would prove the theorized stimulation effect of AM-tACS due to the amplitude modulation of its waveform^26^. We further hypothesize that tACS will induce phosphenes in line with prior studies^48^, but also at lower needed stimulation intensities when compared to AM-tACS^33,34^.

To this day, attempts at in-vivo stimulation with AM-tACS are scarce, and methodological difficulties when using this method remain^37^. This study therefore aims to advance the understanding of AM-tACS and to gain new insights into the feasibility of this method as a new neuromodulatory tool.

## Material and methods

### Subjects

19 healthy subjects (14 female; age range: 19–29 years; mean age: 23.6 years) took part in the experiment. All subjects reported to be free of neurological illness, having no history of epileptic seizures, no metal or other medical implants in their body, had no uncorrected visual impairments and were not taking medication with an effect on the central nervous system. Prior to the experiment, subjects gave written informed consent after being informed about the experimental procedure as well as potential adverse effects of TES. Two subjects had to be excluded from data analysis, because in at least two conditions their phosphene thresholds were substantially higher than those conditions’ mean phosphene thresholds (three standard deviations above the mean), possibly due to a problem with the attachment of the electrodes or non-compliance with the task. This left 17 subjects (12 female; age range: 19–29 years; mean age: 23.8 years) for the data analysis. This research was approved by the local ethics committee of the University Clinic of Magdeburg and carried out in accordance with the guidelines of the Declaration of Helsinki.

### Design and staircase procedure

A trial consisted of three seconds of ramping the stimulation up, five seconds of stimulation^48^, followed by one second of ramping down. A timer on the screen informed subjects when the stimulation was ramped up/down, as well as of the remaining duration of the stimulation. After the stimulation period, subjects answered a self-paced yes/no question if they saw phosphenes during stimulation (using their index and middle finger on the buttons ‘J’ and ‘N’ on a keyboard). Subjects were instructed to keep their eyes open at all times. After subjects gave their response, the next trial started. To avoid condition repetition effects, the order of conditions was pseudorandomized such that each trial was followed by a trial of another condition. This was done to balance carry-over effects across conditions.

Trials were embedded in a condition-wise staircase procedure which determined stimulation intensity for each trial based on prior given responses for trials of that condition. This was done to determine thresholds for phosphene perception for each condition, using a 1-up-1-down staircase procedure^49^. Depending on the subjects’ answer for a trial, the stimulation intensity for its corresponding condition was adjusted, with an increase in stimulation intensity for future trials of that condition if no phosphenes were seen and vice versa. The rate of stimulation intensity adjustment was dependent on the number of reversals of answers. A reversal of answers was counted when a ‘yes’ (i.e., phosphenes were seen) answer to a condition was followed by a ‘no’ (i.e., no phosphenes were seen) answer or vice versa. In the beginning, at zero reversals, intensities were adjusted by 50% of the condition’s stimulation intensity (e.g., a condition set to 1 mA was lowered to 0.5 mA for future trials if it was able to induce phosphenes), with each reversal lowering the adjustment rate by 5%. This meant that after each reversal, the intensity adjustment for a condition became incrementally smaller, decreasing from the initial 50% adjustment rate at the beginning, down to a 15% adjustment rate for trials after the 7th reversal. This allowed for quickly reaching the area of the threshold with larger steps at the beginning, as well as closing in on the precise threshold value with smaller steps at the end.

The staircase for a condition was concluded in one of three possible ways: (1) with the occurrence of an 8th reversal, (2) with the 20th trial in that condition, (3) with the condition running into its maximum intensity value for a 2nd time without phosphene percept. If the staircase concluded in the first or second way, the phosphene threshold was calculated as the mean of all intensities at reversals, excluding the first reversal. If it concluded in the third way, the phosphene threshold for that condition was set to the maximum of 2 mA for statistical analyses.

Every ten minutes of the experiment, subjects took a one-minute fixed break.

### Stimulation

The transcranial electrostimulation was applied using a battery-driven stimulator system (DC-Stimulator Plus, NeuroConn GmbH, Ilmenau, Germany) operating in external mode. The stimulation was driven via a custom Matlab (Version 2019a, Mathworks, Natick, USA) script using the data acquisition toolbox and a digital/analog converter (DAC; NI USB-6212, National Instruments, Austin, TX, USA) connected to the REMOTE port of the stimulator.

For the electrode setup, two circular (34 mm diameter) carbon–rubber conductive electrodes (NeuroConn GmbH, Ilmenau, Germany) were used, with one electrode placed at FpZ and the second electrode vertically centered and approximately 2.5 cm below the right eye (see Fig. 1b). By using relatively small electrodes placed close to the eye, we were able to maximize the current that reaches the retina (for a simulation of current distribution see Fig. 1c). Electrodes were applied to the skin using conductive paste (Ten20, D.O. Weaver, Aurora, CO, USA) with impedances being kept below 5 kΩ.

In this experiment, the maximum stimulation intensity was determined individually for each subject by a pain threshold procedure. Pain thresholds were determined by manually increasing the stimulation intensity of a 200 Hz tACS (the condition at which pain sensations occurred most often), beginning at 0.5 mA peak-to-peak and increasing in steps of 0.25 mA, until subjects either reported adverse side effects (pain sensations or uncomfortable tingling at the electrodes), or the maximum stimulation intensity of 2 mA peak-to-peak was reached. This pain threshold was set as the maximum stimulation intensity for all stimulation conditions except the 1000 Hz conditions (whose possible maximum stimulation intensity was always set to 2 mA), as stimulation with frequencies this high does not induce pain sensations.

Sinusoidal stimulations were applied with no DC offset. Due to a difference in expected phosphene thresholds for tACS and AM-tACS, initial stimulation intensities (i.e., intensities at which the respective staircases started) were set to 0.15 mA for tACS conditions and 1 mA for AM-tACS conditions.

For AM-tACS, the signal was computed based on the following equation:

$$AM_{Signal} \left( t \right) \, = \, a_{stim} *\frac{{\left( {{\text{sin}}\left( {2\pi *f_{c}*t} \right) + {\text{sin}}\left( {2\pi *\left( {f_{c} + f_{m}} \right)*t} \right)} \right)}}{2}$$

where *t* is the time course, *a*_*stim*_ the amplitude of the sine wave, *f*_*c*_ the frequency of the carrier and *f*_*m*_ the frequency of the amplitude modulation. The equation is taken from temporal interference studies^38^ instead of AM-tACS studies^26^ to allow us to also draw conclusions from the results to temporal interference stimulation. An amplitude modulated signal generated via temporal interference differs slightly from AM-tACS waveforms as its envelope is non-sinusoidal (sharp instead of round troughs). This unlikely affected our results, since it only causes a minor difference between the waveforms. To our knowledge there are no studies that make a statement about possible differences in stimulation effects due to the different shape of the waveforms; quite the opposite, they are usually assumed to work in similar ways. Only Kasten et al.^37^ did a direct comparison between both waveforms, but only to confirm that both waveforms were inducing similar artefacts, without making a statement about differing stimulation mechanisms.

The amplitude modulation frequencies (8 Hz, 16 Hz, 28 Hz) were chosen based on tACS findings^48^, to have one frequency (16 Hz) with a very low phosphene threshold, i.e. that optimally induces phosphenes at low stimulation intensity, as well as two less-optimal phosphene inducing frequencies (8 Hz, 28 Hz), with phosphene thresholds matching each other. As for carrier frequencies (50 Hz, 200 Hz, 1000 Hz), one frequency (50 Hz) was chosen to be low enough to induce phosphenes regardless of amplitude modulation, another carrier frequency (200 Hz) was chosen based on an AM-tACS study^36^, while the third (1000 Hz) was based on a study using temporal interference stimulation^38^. This resulted in a combination of 9 different stimulation patterns for AM-tACS (50 × 8, 50 × 16, 50 × 28, 200 × 8, 200 × 16, 200 × 28, 1000 × 8, 1000 × 16, 1000 × 28).

All amplitude modulation and carrier frequencies which were used for AM-tACS, were also administered using tACS (8 Hz, 16 Hz, 28 Hz, 50 Hz, 200 Hz, 1000 Hz). This was done to have tACS as a benchmark to (1) compare stimulation efficacy of amplitude modulation frequencies, when used with tACS vs AM-tACS, (2) to test if carrier frequencies themselves were able to induce phosphenes and (3) to disentangle the effects of amplitude modulation and carrier frequency on phosphene induction.

In order to detect subjects’ tendency for false positive responses (i.e., answering ‘yes’ to having seen phosphenes without actual phosphene perception), three control conditions were included. The first was a sham condition without stimulation. In addition, controls using a transcranial direct current stimulation (tDCS) with anodal (anode above and cathode below the eye) and cathodal (cathode above and anode below the eye) stimulation were used to detect subjects who based their answer on skin sensations caused by the stimulation rather than on phosphene perceptions. The sham stimulation was set to 0 mA. The initial stimulation intensity for the tDCS controls was set to 0.1 mA with a maximum of 0.5 mA.

### Procedure

Subjects were seated in a chair in front of a grey screen (RGB: 50 50 50) in a dimly lit room. After the stimulation was set up, individual pain thresholds were determined for each subject. Following that, subjects were familiarized with phosphene perception by administering a 16 Hz tACS with a stimulation intensity of 0.5 mA. Subjects were asked to verbally describe the shape of perceived phosphenes and the affected visual field, with all subjects reporting flashing lights in the right visual field. Following that, the task was explained to the subjects: after receiving stimulation, subjects were instructed to indicate if they saw phosphenes. At the beginning, subjects performed a training version of the task (5 trials) where 16 Hz tACS using differing intensities (0 mA, 0.125 mA, 0.25 mA, 0.375 mA, 0.5 mA) in a randomized order was administered. If no questions remained, the experiment began, taking approximately 30 min. Afterwards, subjects were reimbursed for their participation either with money or with course credit.

### Statistical analysis

Statistical analyses were performed using SPSS Statistics v22 (IBM, Chicago, IL). Mean phosphene thresholds for each condition were calculated across all 17 subjects and examined using separate repeated measures analyses of variance (ANOVA) for tACS and AM-tACS.

A Shapiro–Wilk test (due to its high power compared to other normal-distribution tests^51,52^) was used to analyse the normal distribution assumption for all conditions that were able to induce phosphenes (TACS: 8 Hz, 16 Hz, 28 Hz, 50 Hz; AM-tACS: 50 × 8, 50 × 16, 50 × 28), with results indicating that all phosphene-inducing conditions except the 8 Hz (*W*(17) = 0.852, *p* = 0.011) and 16 Hz (*W*(17) = 0.784, *p* = 0.001) tACS conditions were normally-distributed. Non-phosphene-inducing conditions (TACS: 200 Hz, 1000 Hz; AM-tACS: 200 × 8, 200 × 16, 200 × 28, 1000 × 8, 1000 × 16, 1000 × 28) were not normally-distributed because they were set to maximum stimulation intensity of 2 mA for statistical analysis as they failed to induce phosphenes and hence showed no variance.

To analyse tACS conditions, a Friedman ANOVA was performed for the factor Stimulation Frequency (8 Hz, 16 Hz, 28 Hz, 50 Hz, 200 Hz, 1000 Hz). A Friedman ANOVA was chosen in this case due to some tACS conditions (200 Hz, 1000 Hz) not being normally distributed. As tACS conditions included only one within-subject factor, the Friedman ANOVA is a suitable non-parametric alternative.

For AM-tACS analysis, a non-parametric ANOVA would be preferable as well due to non-normally distributed AM-tACS conditions, but a non-parametrical approach is not feasible in this case due to more than one within-subject factor. Furthermore, studies argue that the repeated-measures ANOVA is sufficiently robust against non-normal distribution^53–55^. Therefore, AM-tACS conditions were analysed using a repeated-measures ANOVA with the factors Carrier Frequency (50 Hz, 200 Hz, 1000 Hz) and Amplitude Modulation Frequency (8 Hz, 16 Hz, 28 Hz).

Further, to directly compare stimulation methods and analyse differences between tACS and AM-tACS, a two-way repeated measures ANOVA with the factors Stimulation Method (TACS, AM-tACS) and Stimulation Frequency (8 Hz, 16 Hz, 28 Hz) was performed.

Mauchly’s test of sphericity was used to ensure that sphericity could be assumed for all factors, with none violating this assumption. Significant main effects and interactions were followed up using Bonferroni-adjusted post-hoc tests. As an effect size measure, partial eta squared ${(\eta }_{p}^{2}$) is reported for repeated-measures ANOVAs.

For non-significant effects, repeated measures analyses were performed using a Bayesian framework in JASP (JASP Team, Version 0.14.1, 2020) to dissociate the lack of a statistical effect from poor sensitivity to uncover such an effect. Using Bayes analysis, a likelihood ratio of two competing hypotheses – the null hypothesis (H0) and an alternative hypothesis (H1) – is expressed using the factor BF_10_ (probability of the H1 over the H0) or alternatively using the BF_01_ (probability of the H0 over the H1). Bayes factors can range from 0 to infinity, with higher values indicating more support for the hypothesis. For instance, BF_10_ = 2 indicates that the alternative hypothesis is twice as likely as the null hypothesis. For interpretation, a Bayes factor between 1 and 3 is considered weak evidence, up to 10 is considered moderate evidence and Bayes factors above 10 are considered strong evidence^56^.

## Results

All subjects tolerated the stimulation and no serious adverse-effects were reported after the experiment. The only adverse effect reported by subjects was an uncomfortable tingling sensation during stimulation. None of the 17 subjects reported seeing phosphenes during the sham or tDCS-control conditions. All subjects were able to tolerate maximum stimulation intensities above 1 mA [*M* = 1.74 mA, *SD* = 0.30 mA, *Min* = 1.175 mA, *Max* = 2.00 mA] with n = 8 subjects tolerating the maximum stimulation intensity of 2 mA (see Fig. 1d).

### TACS

**Figure 2:**
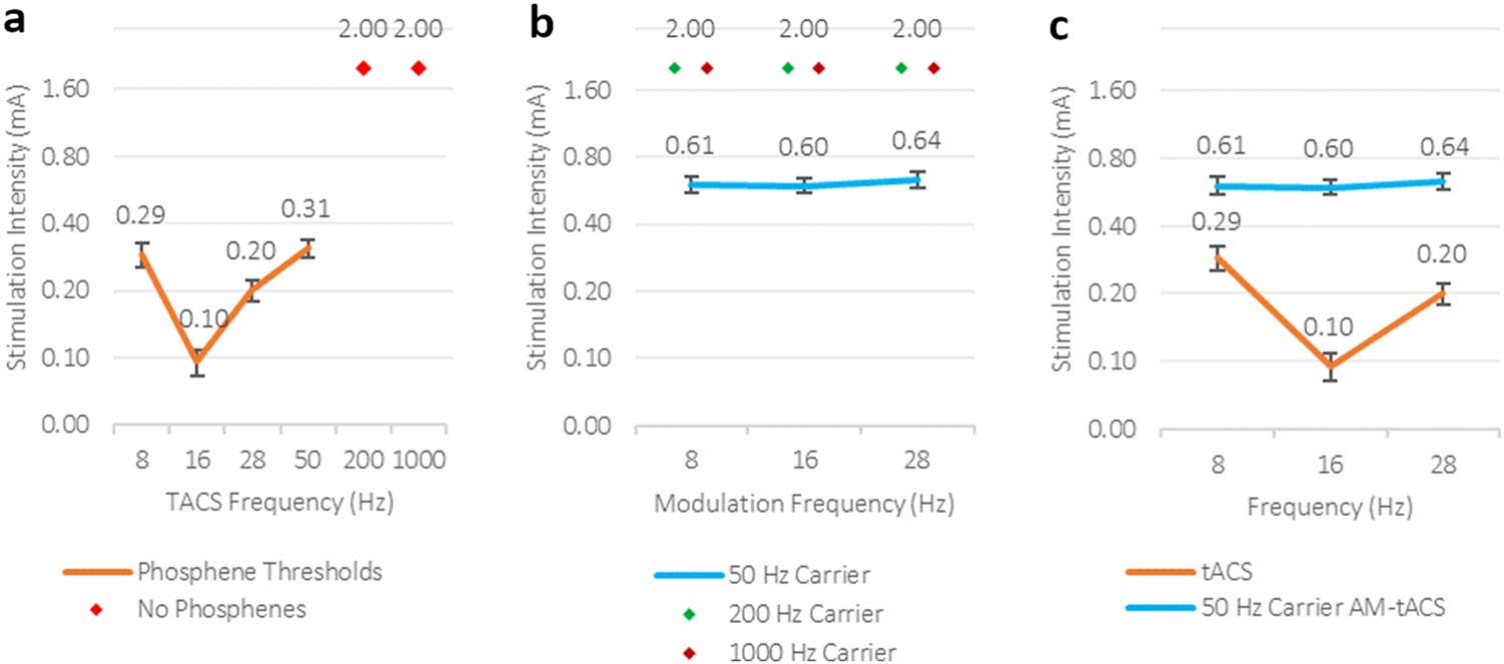
**(a)** Phosphene thresholds for all tACS conditions (8 Hz, 16 Hz, 28 Hz, 50 Hz, 200 Hz, 1000 Hz). Conditions form a V-shaped relation, with 16 Hz needing the least stimulation intensity to induce phosphenes. The 200 Hz and 1000 Hz stimulation did not induce phosphenes and were therefore set to the maximum stimulation intensity of 2 mA. **(b)** Phosphene thresholds for all AM-tACS conditions; 50 Hz carrier conditions (50 × 8, 50 × 16, 50 × 28), 200 Hz carrier conditions (200 × 8, 200 × 16, 200 × 28) and 1000 Hz carrier conditions (1000 × 8, 1000 × 16, 1000 × 28). Phosphene thresholds for the 50 Hz carrier conditions were similar across modulation frequencies. None of the 200 Hz and 1000 Hz carrier conditions induced phosphenes and therefore reached the maximum stimulation intensity of 2 mA. **(c)** Phosphene thresholds for AM-tACS modulation frequencies of 50 Hz carrier conditions (50 × 8, 50 × 16, 50 × 28) and corresponding tACS conditions (8 Hz, 16 Hz, 28 Hz).

A Friedman’s ANOVA for tACS with the factor Stimulation Frequency (8 Hz, 16 Hz, 28 Hz, 50 Hz, 200 Hz, 1000 Hz) revealed a statistically significant main effect [χ^2^ (5, N = 17) = 78.875*, p* < 0.001]. Bonferroni-adjusted post-hoc analysis using the Conover Test revealed 16 Hz stimulation [*M* = 0.10 mA, *SD* = 0.05 mA] having significantly the lowest phosphene threshold, i.e., needing the least stimulation intensity to induce phosphenes, compared to all other frequencies except 28 Hz [*p*_*corr*_ < 0.012]. This was followed by the phosphene thresholds for 28 Hz [*M* = 0.20 mA, *SD* = 0.09 mA], 8 Hz [*M* = 0.29 mA, *SD* = 0.15 mA] and 50 Hz [*M* = 0.31 mA, *SD* = 0.11 mA] which differed not significantly from each other [*p*_*corr*_ = 1] but were significantly lower than the 200 Hz [*M* = 2.00 mA, *SD* = 0.00 mA] and 1000 Hz [*M* = 2.00 mA, *SD* = 0.00 mA] conditions [*p*_*corr*_ < 0.033] (see Fig. 2a). Due to 200 Hz and 1000 Hz conditions not inducing phosphenes and therefore being set to 2 mA, they did not differ significantly from each other [*p*_*corr*_ = 1] (see Supplementary Fig. S1 for single subject data of tACS conditions).

### AM-tACS

A repeated-measures ANOVA with the factors Carrier Frequency (50 Hz, 200 Hz, 1000 Hz) and Amplitude Modulation Frequency (8 Hz, 16 Hz, 28 Hz) revealed a significant main effect of Carrier Frequency [*F*(2,32) = 782.59, *p* < 0.001, ${\eta }_{p}^{2}$= 0.98], but neither the main effect of Amplitude Modulation Frequency [*F*(2,32) = 1.11, *p* = 0.34, ${\eta }_{p}^{2}$= 0.07] nor the interaction between both factors [*F*(4,64) = 1.11, *p* = 0.36, ${\eta }_{p}^{2}$= 0.07] were significant. Bonferroni-adjusted post-hoc analysis revealed a significant difference between the 50 Hz carrier frequency [*M* = 0.61 mA, *SD* = 0.20] and 200 Hz [*M* = 2.00 mA, *SD* = 0.00 mA] as well as 1000 Hz [*M* = 2.00 mA, *SD* = 0.00 mA] carrier frequencies [*p*_*corr*_ < 0.001] (see Fig. 2b). No difference between 200 and 1000 Hz carrier frequencies were found [*p*_*corr*_ = 1], due to no phosphenes being induced in these conditions and their phosphene thresholds reaching the maximum stimulation intensity of 2 mA (see Supplementary Fig. S2 for single subject data of AM-tACS conditions). Using a Bayesian repeated measures ANOVA, there is decisive evidence in favor of an effect of the carrier frequency (BF_10_ = 3.912*10^114^), but also strong evidence for an absence of effect for the amplitude modulation (BF_01_ = 15.615).

To analyse if amplitude modulation frequencies can affect the phosphene threshold of the 50 Hz carrier stimulations (the only AM-tACS conditions with obtainable phosphene thresholds), we exploratively compared the 50 Hz carrier conditions 50 × 8 [*M* = 0.61 mA, *SD* = 0.22 mA], 50 × 16 [*M* = 0.60 mA, *SD* = 0.18 mA] and 50 × 28 [*M* = 0.64 mA, *SD* = 0.23 mA], revealing no differences between phosphene thresholds in these conditions [*p*_*corr*_ > 0.662]. Bayesian analysis revealed moderate evidence for an absence of effect for the amplitude modulation in 50 Hz carrier conditions (BF_01_ = 3.06).

### TACS vs. AM-tACS

To analyse the differences in phosphene induction between tACS and AM-tACS, the AM-tACS conditions that were able to induce phosphenes (50 × 8, 50 × 16, 50 × 28) were compared to the corresponding tACS conditions (8 Hz, 16 Hz, 28 Hz) (see Fig. 2c) using a repeated measures ANOVA with the factors Stimulation Method (tACS, AM-tACS) and Stimulation Frequency (8 Hz, 16 Hz, 28 Hz). This revealed a significant main effect of Stimulation Method [*F*(1,16) = 121.02, *p* < 0.001, ${\eta }_{p}^{2}$= 0.88] due to lower phosphene thresholds in the tACS [*M* = 0.20 mA, *SD* = 0.09 mA], compared to the AM-tACS [*M* = 0.61 mA, *SD* = 0.20 mA] conditions.

Furthermore, the Stimulation Frequency main effect was significant [*F*(2,32) = 14.17, *p* < 0.001, ${\eta }_{p}^{2}$= 0.47] but could be explained by the significant interaction between both factors [*F*(2,32) = 16.94, *p* < 0.001, ${\eta }_{p}^{2}$= 0.51]. This interaction was due to significant differences between frequencies for tACS conditions [*p*_*corr*_ < 0.035] (see tACS section above), but not for AM-tACS conditions [*p*_*corr*_ > 0.662] (see AM-tACS section above).

It should be noted, when using 50 Hz as a carrier frequency [*M* = 0.61 mA, *SD* = 0.20] it needs around double the intensity [*t*(16) = 10.75, *p* < 0.001] to elicit phosphenes as compared to when 50 Hz is used as a tACS frequency [*M* = 0.31 mA, *SD* = 0.11], because 50 Hz AM-tACS, over time, is reduced in amplitude compared to tACS by a factor of 2 due to the amplitude modulation.

## Discussion

In this study we evaluated the efficacy of AM-tACS to gauge its potential as a neuromodulatory tool. To this end, we applied AM-tACS as well as tACS at different stimulation frequencies to the retina of the right eye to induce phosphenes. Our results show that, while tACS clearly induces phosphenes in line with previous studies^48,57^, the amplitude modulation of sine waves, which is the key stimulation mechanism of AM-tACS^26^, did not induce phosphenes. This indicates that AM-tACS does not sufficiently stimulate neurons in the retina which questions its use as a non-invasive neuromodulatory tool.

While the stimulation of the CNS using tACS is a method which has garnered much attention in the last years^2,58–60^, a recent variation of tACS, which uses amplitude modulated sine-waves (AM-tACS)^26^, is only scarcely researched. Thus, in this study, we aimed to evaluate AM-tACS in respect to its neuromodulatory potential and examined the influence of carrier frequency and amplitude modulation frequency, of which the AM-tACS waveform is comprised of. For this, we used the retina as a model for CNS circuitry and phosphenes as an indicator for stimulation efficacy^40,61^. We attached electrodes around the right eye of subjects and applied AM-tACS at different carrier- and amplitude modulation frequencies. In addition, to serve as a benchmark, we applied regular tACS at the same frequencies as well.

For tACS, this resulted in a replication of known interactions^48,57^ between stimulation frequency and needed stimulation intensity to reach the phosphene threshold (the lowest intensity at which phosphenes were induced). The 16 Hz stimulation induced phosphenes most readily and needed the least stimulation intensity to reach its phosphene threshold. This was followed by 28 Hz needing around double the stimulation intensity, as well as 8 Hz and 50 Hz needing three times the intensity compared to 16 Hz stimulation to induce phosphenes. Stimulation with even higher frequencies of 200 Hz and 1000 Hz did not induce phosphenes. This was expected due to the low-pass nature of neurons which greatly reduces the stimulation effectiveness^29^.

For AM-tACS, this low-pass nature of neurons is circumvented by relying not on the high frequency of the sine wave (the carrier frequency) but rather on a much lower-frequency amplitude modulation (modulation frequency) of the sine wave, with the latter being the source of stimulation effects^34^. However, our results show that, within typical tACS intensity limits of 2 mA peak-to-peak, it is not possible to induce phosphenes via the modulation frequency. None of the AM-tACS conditions using 200 Hz or 1000 Hz carrier frequencies were able to induce phosphenes. The only AM-tACS conditions with obtainable phosphene thresholds were conditions using a 50 Hz carrier. However, when comparing tACS at 50 Hz and AM-tACS with a 50 Hz carrier, it became clear that the induced phosphenes in these conditions were not influenced by the amplitude modulation, but rather solely relied on the carrier frequency. In addition, a direct comparison between amplitude modulation frequencies (using the 50 Hz carrier) did not reveal differences in phosphene thresholds.

Furthermore, our results revealed that 50 Hz AM-tACS conditions need around double the stimulation intensity compared to 50 Hz tACS to induce phosphenes. This was expected, since the amplitude modulation reduces the net stimulation intensity to about half. This is a limitation of all studies using amplitude modulated signals and should be considered when interpreting results, as a non-amplitude-modulated signal (e.g., with 1 mA) is only comparable to an amplitude modulated signal with double the intensity (e.g., 2 mA). Consequently, we need to assume that phosphenes in the AM-tACS conditions were only due to the carrier frequency. Thus, it can be concluded that modulation frequencies had no stimulatory effect on the retina and did not induce phosphenes.

There are stimulation techniques, such as transcranial magnetic stimulation (TMS) or electric deep brain stimulation (DBS) which are strong enough to trigger action potentials in stimulated brain cells, thus achieving supra-threshold stimulation. In contrast, non-invasive transcranial electric stimulation techniques such as tACS are generally not strong enough to trigger action potentials. Rather, by using mechanisms such as entrainment or causing changes in synaptic plasticity, tACS uses sub-threshold modulation to facilitate or inhibit a neuron’s likelihood to fire, therefore biasing neuronal activity^62–64^. Phosphenes are a rare example, because they manage to bridge the gap between non-invasive electrostimulation and supra-threshold stimulation, as the cells of the retina are sensitive enough to be triggered by low electric currents. This allows for an easily attainable and reliable readout of stimulation effects.

Though phosphenes have been extensively used as an indicator of neural activation^40,57,61^, it is still not well understood how they are elicited by electric stimulation. As it was first believed that (non-invasively) electrically induced phosphenes were due to direct cortical activation^41^, further studies showed that phosphenes are retinal (and not cortical) in origin^42–47^. For instance, a study from Kar and Krekelberg^47^ debates that stimulation of axons of the retinal ganglion cells are a possible source for phosphenes. The results of this study are supported by a study from Delbeke et al.^65^ who induced phosphenes via direct optic nerve stimulation. This is also in line with studies that show that especially axon terminals are susceptible to electric stimulation^66^. A modeling study^34^ confirmed that the sensitivity to amplitude modulated stimulation using high-frequency carriers is dependent on a fast membrane time constant (i.e., the amount of time it takes for a change in potential from initial value to 63% of its final value). As axons have a very fast membrane time constant^34^, the induction of phosphenes with AM-tACS via axonal stimulation should be theoretically possible.

A limitation of this approach (and therefore our findings) is its generalizability, because stimulation efficacy measures are not directly translatable from retinal cells to brain cells, since stimulation is achieved by different mechanisms. However, a number of studies have shown that tACS causes modulation in brain cells (via sub-threshold modulation using entrainment and changes in synaptic plasticity) as well as an activation of retinal cells (via supra-threshold stimulation), causing an induction of phosphenes. Therefore, it is possible to observe a tACS effect by measuring either of those. Hence, in our study we aimed to establish this link between retinal and brain activation for AM-tACS. However, our results question AM-tACS’ efficacy in retinal stimulation because no supra-threshold stimulation could be achieved. However, this does not rule out potential sub-threshold effects of AM-tACS. Further research in this domain is therefore required.

The question remains why AM-tACS in our study failed to induce phosphenes. One possible explanation for that might be that the intensity of AM-tACS we used was too low to induce phosphenes. Modeling studies have estimated that the needed stimulation intensity of amplitude modulated sine waves has to be multiple times higher than with unmodulated sine waves, to yield comparable stimulation effects^33^. In an empirical study, Esmaeilpour et al.^34^ applied AM-tACS to rodent hippocampal brain slices to show a modulation of neuronal firing during stimulation, but with considerably more stimulation intensity needed than with tACS (at least 167 mA with AM-tACS compared to 2 mA with tACS). As we applied stimulation intensities with a maximum of 2 mA in this study, this may explain why no phosphenes could be induced. One could consider using higher stimulation intensities for AM-tACS, however this would require higher carrier frequencies (which induce less skin sensations), since only half of our subjects were able to tolerate the maximum stimulation intensity of 2 mA without pain sensations when using 200 Hz carriers. But as studies have shown, there exists a trade-off between higher carrier frequencies also needing higher stimulation intensities for comparable effects^34^. Applying higher stimulation intensities is therefore not trivial, since one of the most important features of (AM-)tACS is its (pain-free) non-invasiveness, which cannot just be solved by increasing the carrier frequency.

As, due to reasons discussed here, we were not able to observe supra-threshold activation, future research has to determine if AM-tACS can cause sub-threshold modulations of neural activity instead.

## Conclusion

In this study we evaluated the efficacy of AM-tACS as a neuromodulatory tool, by inducing phosphenes which served as an indicator of neuronal activation. Using a two-electrode setup around the right eye and different carrier- and modulation frequencies, we show that AM-tACS with stimulation intensities of up to 2 mA peak-to-peak is not able to induce phosphenes. While ultimately we did not induce phosphenes using AM-tACS, this study may serve as the basis for future studies that aim to better understand the mechanisms and efficacy of AM-tACS.

## Supplementary Information

Supplementary Information.

## Data Availability

The datasets generated during and/or analysed during the current study are available from the corresponding author on reasonable request.

## References

[1] Başar E, Başar-Eroglu C, Karakaş S, Schürmann M (2001). Gamma, alpha, delta, and theta oscillations govern cognitive processes. Int. J. Psychophysiol..

[2] Vosskuhl J, Strüber D, Herrmann CS (2018). Non-invasive brain stimulation: A paradigm shift in understanding brain oscillations. Front. Hum. Neurosci..

[3] Herrmann CS, Strüber D (2017). What can transcranial alternating current stimulation tell us about brain oscillations?. Curr. Behav. Neurosci. Rep..

[4] Paulus W, Nitsche MA, Antal A (2016). Application of transcranial electric stimulation (tDCS, tACS, tRNS): From motor-evoked potentials towards modulation of behaviour. Eur. Psychol..

[5] Herrmann CS, Rach S, Neuling T, Strüber D (2013). Transcranial alternating current stimulation: A review of the underlying mechanisms and modulation of cognitive processes. Front. Hum. Neurosci..

[6] Herrmann CS, Strüber D, Helfrich RF, Engel AK (2016). EEG oscillations: From correlation to causality. Int. J. Psychophysiol..

[7] Reato D, Rahman A, Bikson M, Parra LC (2010). Low-intensity electrical stimulation affects network dynamics by modulating population rate and spike timing. J. Neurosci..

[8] Reato D, Rahman A, Bikson M, Parra LC (2013). Effects of weak transcranial alternating current stimulation on brain activity—A review of known mechanisms from animal studies. Front. Hum. Neurosci..

[9] Vosskuhl J, Huster RJ, Herrmann CS (2015). Increase in short-term memory capacity induced by down-regulating individual theta frequency via transcranial alternating current stimulation. Front. Hum. Neurosci..

[10] Joundi RA, Jenkinson N, Brittain J-S, Aziz TZ, Brown P (2012). Driving oscillatory activity in the human cortex enhances motor performance. Curr. Biol..

[11] Polanía R, Nitsche MA, Korman C, Batsikadze G, Paulus W (2012). The importance of timing in segregated theta phase-coupling for cognitive performance. Curr. Biol..

[12] Lustenberger C, Boyle MR, Foulser AA, Mellin JM, Fröhlich F (2015). Functional role of frontal alpha oscillations in creativity. Cortex.

[13] Kar K, Krekelberg B (2014). Transcranial alternating current stimulation attenuates visual motion adaptation. J. Neurosci..

[14] Rufener KS, Zaehle T, Oechslin MS, Meyer M (2016). 40Hz-Transcranial alternating current stimulation (tACS) selectively modulates speech perception. Int. J. Psychophysiol..

[15] Marshall L, Helgadóttir H, Mölle M, Born J (2006). Boosting slow oscillations during sleep potentiates memory. Nature.

[16] Riecke L, Formisano E, Herrmann CS, Sack AT (2015). 4-Hz transcranial alternating current stimulation phase modulates hearing. Brain Stimul..

[17] Noury N, Hipp JF, Siegel M (2016). Physiological processes non-linearly affect electrophysiological recordings during transcranial electric stimulation. Neuroimage.

[18] Kasten FH, Dowsett J, Herrmann CS (2016). Sustained aftereffect of α-tACS lasts up to 70 min after stimulation. Front. Hum. Neurosci..

[19] Veniero D, Vossen A, Gross J, Thut G (2015). Lasting EEG/MEG aftereffects of rhythmic transcranial brain stimulation: Level of control over oscillatory network activity. Front. Cell. Neurosci..

[20] Voss U (2014). Induction of self awareness in dreams through frontal low current stimulation of gamma activity. Nat. Neurosci..

[21] Gebodh N (2019). Inherent physiological artifacts in EEG during tDCS. Neuroimage.

[22] Neuling T (2015). Friends, not foes: Magnetoencephalography as a tool to uncover brain dynamics during transcranial alternating current stimulation. Neuroimage.

[23] Soekadar SR (2013). In vivo assessment of human brain oscillations during application of transcranial electric currents. Nat Commun.

[24] Kasten FH, Herrmann CS (2019). Recovering brain dynamics during concurrent tACS-M/EEG: An overview of analysis approaches and their methodological and interpretational pitfalls. Brain Topogr..

[25] Noury N, Siegel M (2017). Phase properties of transcranial electrical stimulation artifacts in electrophysiological recordings. Neuroimage.

[26] Witkowski M (2016). Mapping entrained brain oscillations during transcranial alternating current stimulation (tACS). Neuroimage.

[27] Riddle J, McFerren A, Frohlich F (2021). Causal role of cross-frequency coupling in distinct components of cognitive control. Prog. Neurobiol..

[28] Alekseichuk I, Turi Z, Amador de Lara G, Antal A, Paulus W (2016). Spatial working memory in humans depends on theta and high gamma synchronization in the prefrontal cortex. Current Biol..

[29] Deans JK, Powell AD, Jefferys JGR (2007). Sensitivity of coherent oscillations in rat hippocampus to AC electric fields. J. Physiol..

[30] Esmaeilpour, Z. *et al.* Limited sensitivity of hippocampal synaptic function or network oscillations to unmodulated kilohertz electric fields. *eNeuro* **7** (2020). 10.1523/ENEURO.0368-20.2020 PMC7773889 33328248

[31] Beason RC, Semm P (2002). Responses of neurons to an amplitude modulated microwave stimulus. Neurosci. Lett..

[32] Middleton JW, Longtin A, Benda J, Maler L (2006). The cellular basis for parallel neural transmission of a high-frequency stimulus and its low-frequency envelope. Proc. Natl. Acad. Sci..

[33] Negahbani E, Kasten FH, Herrmann CS, Fröhlich F (2018). Targeting alpha-band oscillations in a cortical model with amplitude-modulated high-frequency transcranial electric stimulation. Neuroimage.

[34] Esmaeilpour Z, Kronberg G, Reato D, Parra LC, Bikson M (2021). Temporal interference stimulation targets deep brain regions by modulating neural oscillations. Brain Stimul..

[35] Chander BS (2016). tACS phase locking of frontal midline theta oscillations disrupts working memory performance. Front. Cell. Neurosci..

[36] Minami S, Amano K (2017). Illusory jitter perceived at the frequency of alpha oscillations. Curr. Biol..

[37] Kasten FH, Negahbani E, Fröhlich F, Herrmann CS (2018). Non-linear transfer characteristics of stimulation and recording hardware account for spurious low-frequency artifacts during amplitude modulated transcranial alternating current stimulation (AM-tACS). Neuroimage.

[38] Grossman N (2017). Noninvasive deep brain stimulation via temporally interfering electric fields. Cell.

[39] Lindenblatt G, Silny J (2002). Electrical phosphenes: On the influence of conductivity inhomogeneities and small-scale structures of the orbita on the current density threshold of excitation. Med. Biol. Eng. Compu..

[40] Attwell D (2003). Interaction of low frequency electric fields with the nervous system: the retina as a model system. Radiat. Prot. Dosimetry..

[41] Kanai R, Chaieb L, Antal A, Walsh V, Paulus W (2008). Frequency-dependent electrical stimulation of the visual cortex. Curr. Biol..

[42] Schwiedrzik CM (2009). Retina or visual cortex? The site of phosphene induction by transcranial alternating current stimulation. Front. Integr. Neurosci..

[43] Schutter DJLG, Hortensius R (2010). Retinal origin of phosphenes to transcranial alternating current stimulation. Clin. Neurophysiol..

[44] Paulus W (2010). On the difficulties of separating retinal from cortical origins of phosphenes when using transcranial alternating current stimulation (tACS). Clin. Neurophysiol..

[45] Evans ID, Palmisano S, Croft RJ (2021). Retinal and cortical contributions to phosphenes during transcranial electrical current stimulation. Bioelectromagnetics.

[46] Laakso I, Hirata A (2013). Computational analysis shows why transcranial alternating current stimulation induces retinal phosphenes. J. Neural Eng..

[47] Kar K, Krekelberg B (2012). Transcranial electrical stimulation over visual cortex evokes phosphenes with a retinal origin. J. Neurophysiol..

[48] Evans ID, Palmisano S, Loughran SP, Legros A, Croft RJ (2019). Frequency-dependent and montage-based differences in phosphene perception thresholds via transcranial alternating current stimulation. Bioelectromagnetics.

[49] Cornsweet TN (1962). The staircase-method in psychophysics. Am. J. Psychol..

[50] Thielscher, A., Antunes, A. & Saturnino, G. B. Field modeling for transcranial magnetic stimulation: A useful tool to understand the physiological effects of TMS? *2015 37*^*th*^* annual international conference of the IEEE engineering in medicine and biology society (EMBC)* **2015,** 222–225 (2015). 10.1109/EMBC.2015.7318340 26736240

[51] Razali NM, Wah YB (2011). Power comparisons of shapiro-wilk, kolmogorov-smirnov, lilliefors and anderson-darling tests. J. Stat. Model. Anal..

[52] Steinskog DJ, Tjøstheim DB, Kvamstø NG (2007). A cautionary note on the use of the Kolmogorov–Smirnov test for normality. Mon. Weather Rev..

[53] Blanca MJ, Alarcón R, Arnau J, Bono R, Bendayan R (2017). Non-normal data: Is ANOVA still a valid option?. Psicothema.

[54] Schmider E, Ziegler M, Danay E, Beyer L, Bühner M (2010). Is it really robust?. Methodology.

[55] Salkind, N. J. *Encyclopedia of Research Design* (Sage, 2010).

[56] van Doorn J (2021). The JASP guidelines for conducting and reporting a Bayesian analysis. Psychon. Bull. Rev.

[57] Turi Z (2013). Both the cutaneous sensation and phosphene perception are modulated in a frequency-specific manner during transcranial alternating current stimulation. Restor. Neurol. Neurosci..

[58] Yavari F, Jamil A, Mosayebi Samani M, Vidor LP, Nitsche MA (2018). Basic and functional effects of transcranial Electrical Stimulation (tES)-An introduction. Neurosci. Biobehav. Rev..

[59] Antal A, Paulus W (2013). Transcranial alternating current stimulation (tACS). Front. Hum. Neurosci..

[60] Thut G, Schyns PG, Gross J (2011). Entrainment of perceptually relevant brain oscillations by non-invasive rhythmic stimulation of the human brain. Front. Psychol..

[61] International Commission on Non-Ionizing Radiation Protection (2010). Guidelines for limiting exposure to time-varying electric and magnetic fields (1 Hz to 100 kHz). Health Phys..

[62] Bikson M, Rahman A (2013). Origins of specificity during tDCS: anatomical, activity-selective, and input-bias mechanisms. Front. Hum. Neurosci..

[63] Fertonani A, Miniussi C (2017). Transcranial electrical stimulation: what we know and do not know about mechanisms. Neuroscientist.

[64] Ruhnau P, Rufener KS, Heinze H-J, Zaehle T (2018). Sailing in a sea of disbelief: in vivo measurements of transcranial electric stimulation in human subcortical structures. Brain Stimul..

[65] Delbeke J, Oozeer M, Veraart C (2003). Position, size and luminosity of phosphenes generated by direct optic nerve stimulation. Vision. Res..

[66] Chakraborty D, Truong DQ, Bikson M, Kaphzan H (2018). Neuromodulation of axon terminals. Cereb. Cortex.

