# Supplementary material for: Amplitude modulated transcranial alternating current stimulation (AM-TACS) efficacy evaluation via phosphene induction
Source: Sci Rep. 2021 Nov 15;11:22245. doi: 10.1038/s41598-021-01482-1 (PMC8593032; doi:10.1038/s41598-021-01482-1)
Supplement: Supplementary file 1 — Supplementary Information. [file 41598_2021_1482_MOESM1_ESM.docx]

# Supplement


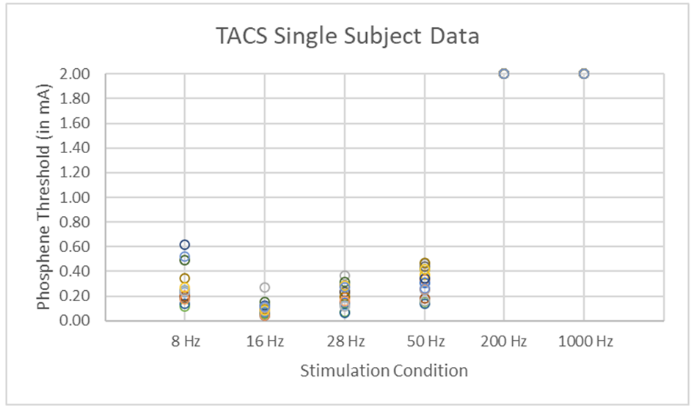


*Figure S1.* Single subject data for tACS conditions.


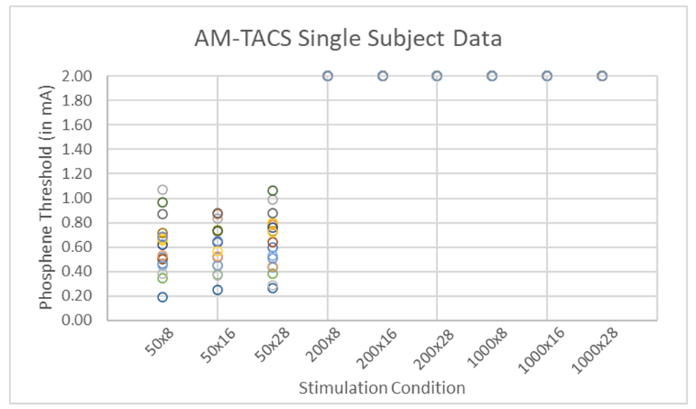


*Figure S2.* Single subject data for AM-tACS conditions.
